# Caregivers’ Compliance and Perception of Daycare Centers—A Community-Based Childhood Drowning Prevention Intervention Implemented in Rural Bangladesh

**DOI:** 10.3390/ijerph19159537

**Published:** 2022-08-03

**Authors:** Lamisa Ashraf, Priyanka Agrawal, Aminur Rahman, Md. Al Amin Bhuiyan, Shumona Sharmin Salam, Qingfeng Li, Abdulgafoor M. Bachani

**Affiliations:** 1 Johns Hopkins International Injury Research Unit, Department of International Health, Johns Hopkins Bloomberg School of Public Health, 615 N Wolfe Street, Baltimore, MD 21205, USA; lashraf1@jhu.edu (L.A.); pagrawa6@jhu.edu (P.A.); qli28@jhu.edu (Q.L.); 2 Centre for Injury Prevention and Research, Bangladesh (CIPRB), House # B-120, Road # 07, New DOHS, Mohakhali, Dhaka 1206, Bangladesh; aminur@ciprb.org (A.R.); al-amin@ciprb.org (M.A.A.B.); 3 International Center for Diarrheal Disease Research, Bangladesh, 68, Shaheed Tajuddin Ahmed Sarani Mohakhali, Dhaka 1212, Bangladesh; shumona.sharmin@gmail.com

**Keywords:** daycare centers, compliance, drowning, children, low-and middle-income countries, Bangladesh

## Abstract

Drowning was one of the most prevalent causes of death worldwide for children under five in 2020. Drowning was the second leading cause of death for children under five in Bangladesh, while 58% of all deaths in the 1–5 years old age group resulted from drowning. Adult supervision helps prevent child drowning in rural areas where water bodies are easily accessible and located very close to homes. This paper aims to assess caregivers’ compliance and perception of community daycare centers in rural Bangladesh, piloted as a child drowning prevention intervention. In this longitudinal study, each child enrolled in the daycare intervention was visited and data on compliance and satisfaction with the daycare were collected. Descriptive statistics on daycare attendance, patterns of supervision, and caregivers’ perceptions about daycare were reported. When inquired about daycare attendance (*n* = 226,552), a total of 77.4% of children (*n* = 175,321) were found to attend daycare. The distance from homes and an adult’s unavailability to take the child to daycare were the most common reasons for not attending or discontinuing enrollment. The majority of children (76.4%) were supervised by their mothers during daycare closures. A total of 67.7% of respondents perceived daycare to be a safe place, where children also developed cognitive (51.7%) and social skills (50.6%). There were no incidences of drowning among children while attending daycare. Rural families were found to be compliant with the daycare and professed it to be a safe place protecting children from drowning and other injuries, while allowing them to focus on household chores or income-generating activities. These findings indicate a potential for the expansion of this intervention in rural Bangladesh and similar settings.

## 1. Introduction

Drowning is one of the leading causes of deaths for children under five years of age, both in high-income and low- and middle-income countries (LMICs) [1]. Between 2017 and 2018, drowning accounted for 58% of deaths among children 1–5 years of age and was listed as the second leading cause of under-five child mortality in Bangladesh [2,3]. In high-income countries (HICs), enactment and enforcement of legislation around the safe use of private and public water bodies as well as provisions for safe and supervised spaces for young children have reduced the burden of drowning over the past couple of decades [1,4]. However, the translation of evidence-based effective interventions to prevent drowning has not seen a similar fate in LMICs; cultural, societal, and geographic dissimilarities towards drowning hinder the head-on implementation of such interventions [5]. This is evident from the fact that the rate of drowning in LMICs in Africa and Asia is 10 to 20 times more than that in the United States [6]. Successful intervention strategies from HICs may not be applicable in LMICs since drowning occurs in day-to-day settings in LMICs, while it is associated with recreational activities such as swimming in HICs [7].

There is some evidence to show that drowning-related deaths among children in LMICs usually occur during the morning and early afternoon hours of the day when parents and caregivers are engaged in household or wage-earning activities. Drowning events mostly occur very close to the home setting, usually within 20 m from the house [8]. Accounting for the circumstantial evidence around drowning deaths, the Johns Hopkins Bloomberg School of Public Health in collaboration with the Center for Injury Prevention and Research, Bangladesh (CIPRB) and the International Center for Diarrheal Disease and Research, Bangladesh (icddr,b) implemented a large-scale population-based study in 2013 to assess the impact of community daycare centers in reducing the risk of drowning among children under five years of age in rural Bangladesh.

The daycare centers were free-for-service and were managed by trained creche mothers and assistants, who provided supervision to children six days a week. Children were engaged in semi-structured activities that involved playing, singing nursery rhymes, becoming familiar with alphabet letters and numbers, and learning good hygiene habits while in the daycare centers. Continuous surveillance of all population in the region showed that the daycare centers were effective in reducing the risk of drowning among children under five years of age [9]. In addition to drowning prevention, the community daycare centers provided additional benefits, for example, a cognitive study found long-term exposure to community daycare centers to result in improved psychosocial developmental outcomes among children 9–17 months of age [10].

As a first of its kind study to evaluate the large-scale effectiveness of the daycare intervention for drowning prevention, the promising results present a huge potential for the expansion and implementation of community daycare centers in Bangladesh and similar countries in the region [10]. The government of Bangladesh is currently working with a technical advisory group to review processes for the expansion of the daycare intervention at the national level [11,12]. Additional research has shown that daycare centers have the potential of saving 10,000 lives globally among its poorest billion [13]. Given these initiatives towards long-term sustainability of the intervention, it is important to understand the perceptions of the host communities towards the daycare centers and their compliance with the intervention. It is known from prior research that active involvement of community members in the process of implementation of community-based interventions leads to smoother uptake of the same [14,15]. Better compliance to an intervention requires the users to understand and acknowledge the benefits and appropriateness of the intervention [16]. This paper describes caregivers’ compliance, and factors that influenced compliance with the daycare intervention in rural Bangladesh as well as the perceptions of parents and caregivers towards the daycare centers.

## 2. Methods

### 2.1. Study Setting, Procedure, and Implementation

The Saving of Lives from childhood Drowning (SoLiD) program, which consisted of a package of interventions—daycare centers and playpens—for childhood drowning prevention, was implemented across seven purposively selected sub-districts in rural Bangladesh (namely, Matlab North, Matlab South, Daudkandi, Chandpur Sadar, Raiganj, Sherpur Sadar, and Manohardi) in 2013 [17,18]. The rationale for selecting these sub-districts for the rollout of the program as well as other methodological details have been explained in earlier research [19]. The package of interventions included daycare centers, locally called ‘anchal’, where children aged 9–47 months were supervised from 9:00 a.m. to 1:00 p.m. by trained ‘creche mothers’ and assistants [9]. In the daycare, children engaged in activities which stimulated their cognitive, physical, social, and linguistic skills. The activities were coordinated by creche mothers and their assistants [10].

Community sensitization via engagement of local stakeholders in the program was performed to raise awareness and educate communities about the burden of drowning and other injuries and related prevention strategies. A baseline census was conducted in 2013 to collect information on socio-demographic characteristics, health-seeking behavior, and injury events from the entire population residing in the 51 unions of the seven selected sub-districts [19]. Following this, a demographic and injury surveillance system was established to monitor the occurrence of major health events, including drowning and other injury deaths among children, for the two-year implementation phase of the program (2013–2016) [9,19]. Additionally, to assess compliance with the daycare intervention, regular follow-up visits were made to households at intervals of two months and four months, alternately. Data collection and implementation activities were led by two local organizations, CIPRB and icddr, b [9]. This study utilizes data collected from the follow-up visits to assess compliance of families and their children with the daycare intervention and their perceptions about the intervention. 

### 2.2. Data Collection 

During the implementation phase of the program, trained data collectors visited homes of children enrolled in the daycare centers and collected data from parents or caregivers on socio-demographic features and daycare attendance, such as current attendance (yes/no), frequency of attendance in the past month, reasons for sending or never sending children to daycare, and reasons for discontinuing daycare attendance. Respondents were also asked about the type of injuries, if any, the children sustained while at the daycare and who supervised them when daycare centers were closed or on days they did not attend. Data on time spent (in hours) on household chores and income generating activities (if any) were also collected. Using a four-point Likert scale, the perceptions of respondents towards daycare centers were also gauged on a pre-designed set of statements that ranged from positive to negative towards the daycare and related activities (e.g., “daycare is a safe place for child”, “daycare helps develop child’s language”, “daycare is not helpful for physical growth of child”, and so on). These follow-up visits were made every two to four months, and data on perception and satisfaction with daycare were only collected every four months. At least four visits were made to each household where a child enrolled in a daycare lived. The data collectors used a short form (without questions on perception and satisfaction) and a long form (with questions on perception and satisfaction) every two and four months, respectively. 

### 2.3. Data Analysis

The datasets on compliance follow-up visits from the two implementing organizations (CIPRB and icddr, b) were combined for analytical purposes. The data were arranged in a longitudinal format and the denominator for all analyses was the average number of children visited across all five follow-up rounds.

Sex of the respondents and children and current daycare attendance were coded as binary variables, while ages of respondents and children were coded as continuous variables. Occupation of the respondent and relation with the child were coded as nominal categorical variables. Variables on frequency of daycare attendance, supervision, reasons for sending and never sending to daycare, and discontinuing daycare were coded as nominal categorical variables. Number of hours spent on income generating activities and household chores were originally coded as a discrete variable with counts from 0–15 h which were separately converted to an ordinal categorical variable with four categories (not engaged, 1–4 h, 5–8 h, and 9–15 h). Data on perception and satisfaction were recorded and labelled on a four-point Likert scale with the following options: strongly agree, agree, disagree, and strongly disagree. 

Descriptive statistical analyses were conducted to describe daycare attendance, patterns of supervision, and perceptions and satisfaction among parents, using the variables of interest. Measures were reported in frequency and percentages, and Chi-squared test was used to show the statistical significance of trends in daycare attendance with each follow-up visit and time spent on income generating activities. All frequencies and percentages are presented as an average across the five follow-up compliance rounds. Stata/MP 16.1 (StataCorp LLC, College Station, TX, USA) was used for all statistical analyses [20]. 

## 3. Results

### 3.1. Characteristics of the Respondents

A total of 67,232 children between the ages of 9 and 60 months were enrolled in the daycare intervention over the two-year project period. Households of children enrolled in the daycare received up to five follow-up visits depending on when they were enrolled in the daycare during the study period. Over the project period, a total of 226,565 visits were made across the five follow-up rounds. The majority of the respondents were the children’s mothers, the primary caregiver in the household (*n* = 219,664; 97.3%) (Table 1).

### 3.2. Daycare Attendance

**Figure 1:**
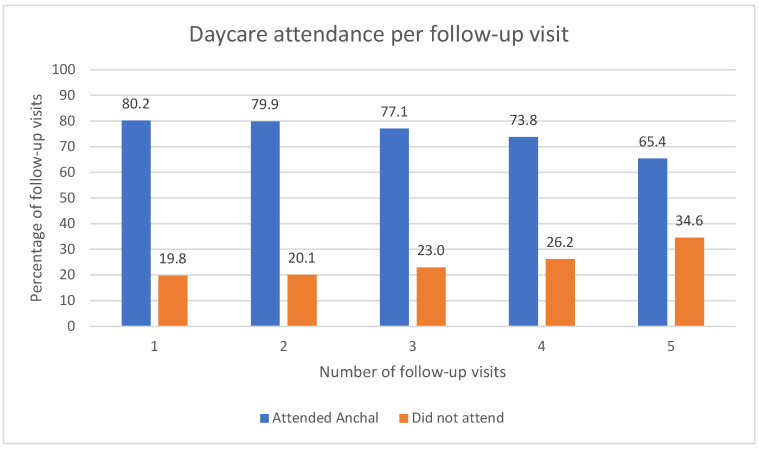
Percentages of daycare attendance per follow-up visit.

Around 77.4% of children (*n* = 175,321) on average had been attending the daycare at the time of a follow-up visit. There were small but statistically significant decreases in the proportion of children attending a daycare with each follow-up visit (*p* < 0.005) (Figure 1).

Of all the children who were attending a daycare, 65.9% (*n* = 115,471) on average attended regularly for more than 20 days in a month, while 25.4% (*n* = 44,444) attended for 7 to 20 days in a month. Around 8.5% of children (*n* = 15,421) on average had infrequent attendance for less than seven days a month.

Of the children who attended daycare regularly, 64.1% (*n* = 55,989) were males. Overall, about 35.0% (*n* = 61,847) of the children who attended were 2–3 years old, and about 32.0% (*n* = 56,596) were 3–4 years old. Daycare attendance was much lower in children under two years of age and for those four years and older. Attendance was lower during the monsoon season, starting from the month of July, and remained low until the winter months (Supplementary Materials, Figure S1a,b).

When respondents were asked about why they preferred sending their children to a daycare, around 81.5% of the respondents reported that they considered the daycare to be a safe space for their children. Around 64.4% said that children could learn reading and writing skills, 54.3% said children could play, and 52.2% responded that children would learn to dance, sing, and learn and recite rhymes. Almost 67.0% mentioned that they could stay anxiety-free, while about 62.0% mentioned that they could focus on their household chores while children were at the daycare (Table 2). During daycare closures over the weekends or on holidays, the children were supervised mainly by the mother (*n* = 172,973, 76.4%) or a grandparent (*n* = 27,614, 12.2%).

Injuries were reported among 0.5% of all children who had ever attended a daycare during the study period. While in the daycare, falls (33.1%) and cuts (22.7%) were the most reported injury types. Respondents reported that around 11.5% of the injuries resulted from scuffles or quarrel among children in the daycare; 63.0% of these were among children two to four years of age. No incidences of drowning were reported.

For children who discontinued going to a daycare after initial enrollment and attendance, respondents cited the distance to the daycare (43.2%), lack of a responsible adult to accompany the child to the daycare (28.8%), and the closure of a daycare center (20.6%) as the main reasons for discontinuity. A very small percentage of the respondents (0.9%) did not see any benefits of having their children attend a daycare (Table 3). The children who discontinued going to a daycare were supervised either by their mothers, aunts, or uncles.

### 3.3. General Perception and Satisfaction towards Daycare

When asked whether the daycare was a safe place, 67.7% of the respondents agreed to the statement. Similarly, respondents agreed that the daycare was helpful in developing language of children (52.4%) and cognition (51.7%), useful in developing child’s morale (51.9%), remove shyness and scariness (51.5%), as well as provide knowledge regarding drowning prevention (52.4%) (Supplementary Materials, Table S1).

Respondents agreed that children attended the daycare with pleasure (51.1%) and learned to socialize with other children and peers (50.6%). Around half of the respondents on average agreed with the statement that parents can also learn many things from children who attended daycare. Respondents believed that the children did not get into trouble at the daycare among each other (91.7%) and did not face difficulties with using the restroom (90.3%), eating (98.4%), or at rest time (94.4%) (Supplementary Materials, Table S1).

Overall, the environment of the daycare was considered good by almost 97.0% of all respondents. Most respondents (97.8%) believed that the supplies, such as toys and books, available in the daycare for children to use were suitable for their age. The daycare was considered as a space that taught children not only to sing and dance but also learn to read and write. Most respondents disagreed with the statement that children were being verbally or physically abused by the daycare supervisors. Respondents believed that all children were treated equally (98.7%) by the daycare supervisors irrespective of their socio-economic status and that the management of children was good (97.6%) (Supplementary Materials, Table S1).

When asked whether the daycare was not conducive for the physical growth of a child, about 95.0% of the respondents on average disagreed with the statement. While almost half (48.3%) of the respondents agreed that leaving children at the daycare gave them relaxation, many respondents (61.5%) had some difficulty with dropping off and picking children from the daycare (Supplementary Materials, Table S1).

### 3.4. Respondents’ Engagement in Income-Generating Activities and Household Chores

Around 10.0% (*n* = 11,059) of the respondents were involved in income generating activities ranging from one hour to 15 hours while their children attended the daycare. An overwhelming majority of those 10.0% were mothers (*n* = 10,167; 91.0%, *p* < 0.001) who were also the primary caregiver or supervisor for their children. They were able to engage in up to four hours of income generating activities during the hours of daycare operation. It was also seen that in a household, the female head, i.e., the mother (*n* = 127,240, 98.3%), was overwhelmingly responsible for household chores, spending on average five to eight hours each day, compared to other family members.

## 4. Discussion

Overall, there is limited past research to show compliance with daycare interventions and its association with drowning or other injury prevention in rural communities of LMICs [21]. The results from this study showed that most primary supervisors for children under five years of age, were mothers; the majority were either unemployed, retired or housewives. This is in line with the findings of another study in which rural families acknowledged the need for adult supervision for children and preferred the mothers to be supervisors [22]. However, prior studies have shown that unemployed caregivers or housewives remain engaged with household chores during the day and are unable to provide constant supervision to young children [8].

More than half of the children enrolled in the daycare intervention attended the daycare centers for more than 20 days in a month. Daycare attendance was higher among children who were two to four years of age, and much lower among younger children (under two years) and older children (over five years). The distance from respondents’ homes and unavailability of an adult to take a child to a daycare were identified as major barriers to attending or continuing to attend daycare. Similarly, distance from homes hindered continuation of daycare attendance in another study from Bangladesh [23]. Month by month analysis of percentage of daycare attendance shows that non-attendance was particularly higher in the colder and monsoon months. This has implications for the effectiveness of daycare interventions for drowning prevention since prior studies have found that childhood drowning peaks during the monsoon season [22].

Parents and caregivers considered the daycare to be a safe space where children gained cognitive and social skills, while remaining protected from the risk of drowning and other injuries, which is consistent with the findings from existing studies in Bangladesh [10,24]. Findings from this study show very few injuries while children remained in daycare.

Sending children to a daycare during regular school hours provided mothers with the opportunity and time to engage in income generating activities as well as to complete household chores without having to worry about their child’s safety. Similarly, another study showed that parents felt stress-free while their children remained at daycare [24]. In the same study, parents reported that their children were eager to attend daycare and that daycare helped prepare children for future formal schooling, a reflection shared by parents in the current study as well [23,24].

Respondents in our study felt that children were learning to read and write and were satisfied with the quality of the toys and books used at daycare. In contrast, in another study from Bangladesh, parents perceived activities at daycare to be repetitive and shared that their children remained hungry and had to use damaged toys while at daycare [23].

The success of a community-based intervention aiming at behavior change depends largely on its ability to convince the community of the risk of health problems while demonstrating the effectiveness of the intervention in removing or reducing the problem [25]. Parents and caregivers of children enrolled in the daycare intervention of the SoLiD program perceived the daycare as a safe place for children where they were safe from the risk of drowning, thus the program succeeded in transferring the message of the importance of adult supervision of children under five, especially during the busier hours of the day when parents or caregivers are unable to supervise children. There were no incidences of drowning among children enrolled in the daycare intervention and few occurrences of injuries at daycare across all follow-up visits which attests to the effectiveness of the intervention. Additionally, a formal systematic evaluation of the daycare intervention in the SoLiD project revealed that the intervention was effective in reducing the risk of drowning from children enrolled in the daycare centers versus those who received another intervention or no intervention at all [9].

Another criterion for a successful community-based intervention is one that involves stakeholders in the intervention’s development and implementation [25]. In the case of the SoLiD program, community sensitization [18] and regular engagement with the community members while focusing on regular and continued functioning of the daycares provided the much-needed background for parents and caregivers to perceive the daycare as a beneficial intervention. Additionally, engagement of the local women in the centers as creche mothers and assistants resulted in a huge buy-in from the community and played a key role in the success of the program [23,25,26].

While the rural community in this study was compliant with the daycare intervention, it is important to note that the daycare provided services free of cost. Additionally, a different rural community in Bangladesh may not be as compliant with the daycare intervention as found in this study [23]. Prior studies have shown that while the daycare model can be affordable for most families in rural Bangladesh, some low-income families might need additional subsidies to partake the benefits of the daycare, a potential area to expand on with support from governmental and non-governmental organizations in Bangladesh [24].

### Limitations

While the study is one of a kind in providing compliance estimates for use of daycare in a rural LMIC setting, it does not come without limitations. While the instrument for collecting data on perception and satisfaction about daycare may have increased accuracy and minimized bias by using a mix of negative and positive statements with responses spread out across a four-point Likert scale, the answers to questions were limited to the options listed on the questionnaire [27]. Since the questions asked were not open-ended, important details on why respondents agreed or disagreed with the statements on perception and satisfaction and further reasons for sending children to daycare, or never attending daycare, for example, may have been missed. In addition, certain results found in this study may have been circumstantial, such as, most respondents whose children attended daycare were unemployed, retired, or housewives. Finally, since the study was conducted in rural areas of specific districts in Bangladesh, the results may not be generalizable to urban areas and other rural areas where compliance and satisfaction with the daycare intervention may vary [23]. Additionally, while we had some information on the amount of time respondents were able to spend on income generating activities, there was no information on the kinds of activities that the respondents were able to engage in. Having this information could have provided some robust analysis on other supplemental programs and policies to increase the impact of daycare centers, not only on injury prevention but also on other facets for involved communities. Lastly, while we had data on injuries sustained by children at daycare, we did not collect information about injuries occurring outside of the daycare setting. This information could have allowed us to explore associations between compliance with daycare centers and incidence of injuries occurring in other settings (e.g., homes, neighborhoods, etc.). It is also important to note that while the daycares were highly acceptable as a drowning prevention intervention within the communities, the implementation of the daycare across the seven sub-districts did not come without its set of challenges which have been discussed elsewhere [9].

## 5. Conclusions

The longitudinal data on compliance and community perception collected in this study can be used to infer long-term sustainability of the daycare intervention and the potential for expansion of the intervention. The importance of community daycare centers for drowning prevention in rural areas of LMICs is becoming increasingly evident with the WHO, in its latest guideline, recommending the daycare intervention for children under six years of age in countries with a high prevalence of drowning [28]. Additional benefits of reduced overall injury rates and improved cognitive skills among children, as well as opportunities for caregivers to spend more time on income-generating work have been reported. Parents and caregivers perceived daycare centers as necessary and beneficial for both them and their children and were compliant with the intervention. The results of this study could be used to develop and implement policies for the establishment of community daycare centers as a drowning prevention strategy with additional focus on early childhood development, in rural Bangladesh, which could potentially serve as a model intervention for countries in the same region with a high prevalence of child drowning. Community sensitization around the daycare intervention would determine effective scale-up.

## Figures and Tables

**Table 1:** Socio-demographic characteristic of respondents for the follow-up visits.

Characteristics of Respondents in Each Follow-up Visit						
	Number of Respondents, *n* (%)					
	1st *N* = 67,232	2nd *N* = 59,806	3rd *N* = 48,058	4th *N* = 34,824	5th *N* = 16,645	Total
**Relation with child**						
Mother	64,734 (96.7)	57,893 (97.2)	46,740 (97.6)	33,981 (98.0)	16,316 (98.3)	219,664 (97.3)
Father	444 (0.7)	328 (0.6)	196 (0.4)	133 (0.4)	42 (0.3)	1143 (0.5)
Sibling	112 (0.2)	83 (0.1)	66(0.1)	40 (0.1)	21 (0.1)	322 (0.1)
Other	1673 (2.5)	1264 (2.1)	873 (1.8)	529 (1.5)	218 (1.3)	4557(2.0)
**Occupation**						
Agriculture	485 (0.7)	387 (0.7)	245 (0.5)	150 (0.4)	38 (0.2)	1305 (0.6)
Business	248 (0.4)	173 (0.3)	114 (0.2)	85 (0.3)	16 (0.1)	636 (0.3)
Student	68 (0.1)	49 (0.1)	38 (0.1)	17 (0.1)	6 (0.0)	178 (0.1)
Housewives ^1^	64,562 (97.0)	57,741 (97.2)	46,496 (97.4)	33,805 (97.8)	16,310 (99.1)	218,914 (97.4)
Service	806 (1.2)	678 (1.1)	534 (1.1)	342 (1.0)	80 (0.5)	2440 (1.1)
Skilled labor	167 (0.3)	140 (0.2)	111 (0.2)	65 (0.2)	10 (0.1)	493 (0.2)
Unskilled labor/Domestic worker	169 (0.3)	143 (0.2)	126 (0.3)	56 (0.2)	4 (0.0)	498 (0.2)
Transport workers	8 (0.0)	7 (0.0)	3 (0.0)	3 (0.0)	0 (0.0)	21 (0.0)
Others	80 (0.1)	60 (0.1)	47 (0.1)	43 (0.1)	3 (0.0)	233 (0.1)

N represents the total number of follow-up visits per round; ^1^ the category ‘housewives’ also includes those who were retired and unemployed, each of whom account for about 0.1% of the respondents in this category.

**Table 2:** Reasons for sending a child to a daycare.

	Across All Follow-ups	
Reason for Sending Child to Daycare ^1^	Frequency (n) *n* = 175,321	Percentage
Child can stay safe	142,846	81.5%
Remain tension free	116,307	66.3%
Can learn reading/writing	112,973	64.4%
Can do household chores	108,489	61.9%
Can play	95,237	54.3%
Can learn to dance/sing/recite/rhymes	91,573	52.2%
Can learn cleanliness from daycare	49,237	28.1%
Can learn manners and etiquette	48,470	27.7%
Nobody else to supervise the child	7394	4.2%
Other children attended	4709	2.7%
Request from daycare supervisor	3831	2.2%
Mother can work outside for money	3404	1.9%
Neighbor’s suggestion	2871	1.6%

^1^ Multiple responses were allowed per respondent.

**Table 3:** Reasons for discontinuing daycare as stated by respondents ^1^.

Reasons	Frequency (*n* = 12,124)	Percentage (%)
***Factors related to children***		
Child does not like being without family members	1377	11.4
Child terrified of attending daycare	342	2.8
Child remains sick	281	2.3
Child becomes tired	137	1.1
Child is too young to go to daycare	95	0.8
Child remains hungry at daycare	80	0.7
Child was injured at daycare	43	0.4
Child is disabled	26	0.2
***Factors related to daycare***		
Too far	5243	43.2
Daycare shut down/opens irregularly/creche mother dropped out	2502	20.6
Creche mother/assistant could not manage/hit the child	127	1.1
Daycare is beside a pond	37	0.3
***Factors related to families***		
No one available to take child to daycare (due to illness, family problems, etc.)	3496	28.8
Temporarily staying/visiting or moving out from the area	358	3.0
Conflict with work	234	1.9
No benefits to attending daycare	107	0.9
Stay at home mother can supervise	38	0.3
***Miscellaneous factors***		
Alternative nearby ^a^	801	6.6
Problem with transport during rainy season	798	6.6
Weather: cold/rain	199	1.6
Others ^b^	258	2.1

^1^ Respondents included parents, siblings, grandparents, aunts, and uncles; ^a^ alternatives include religious teaching institutions/schools/centers for mass education; ^b^ includes children who died from drowning (*n* = 3), from other causes (*n* = 3), and those who graduated (*n* = 34).

## Data Availability

The data presented in this study are available on request from the corresponding author. The data are not publicly available to ensure data security.

## Supplementary Materials

The following supporting information can be downloaded at: https://www.mdpi.com/article/10.3390/ijerph19159537/s1, Table S1: General perception of and satisfaction with daycare among parents whose children were enrolled in a daycare; Figure S1: (a) Variation in daycare attendance by month in 2014, (b) Variation in daycare attendance by month in 2015.
