# Supplementary material for: Caregivers’ Compliance and Perception of Daycare Centers—A Community-Based Childhood Drowning Prevention Intervention Implemented in Rural Bangladesh
Source: Int J Environ Res Public Health. 2022 Aug 3;19(15):9537. doi: 10.3390/ijerph19159537 (PMC9368301; doi:10.3390/ijerph19159537)
Supplement: Supplementary file 1 [file ijerph-19-09537-s001.zip › ijerph-1788084-supplementary.pdf]

Supplementary Material, Table S1: General perception of and satisfaction with daycare among parents whose children were enrolled in a daycare

	Number of visits (*n*, %)				
**Statements**	**Strongly agree**	**Agree**	**Disagree**	**Strongly disagree**	**Total visits**
Daycare is a safe place for child	36,200 (28.5)	49,733 (39.2)	8,955 (7.1)	31,988 (25.2)	126,876
Daycare helps develop child's language	25,316 (20.0)	41,051 (32.4)	34,407 (27.2)	25,904 (20.5)	126,678
Daycare is not helpful for physical growth of child	2,289 (1.8)	3,147 (2.5)	82,580 (65.3)	38,406 (30.4)	126,422
Daycare is useful for developing child's moral	21,108 (16.7)	44,488 (35.2)	34,935 (27.6)	25,869 (20.5)	126,400
Helps reduce shyness and remove fear	25,523 (20.2)	39,541 (31.3)	33,670 (26.6)	27,711 (21.9)	126,445
Children can learn about drowning prevention at daycare	27,300 (21.6)	38,861 (30.8)	36,961 (29.2)	23,265 (18.4)	126,387
Prepares children for future schooling	29,703 (23.6)	34,145 (27.1)	29,524 (23.4)	32,648 (25.9)	126,020
Child stays in daycare with pleasure	31,353 (24.8)	33,241 (26.3)	20,707 (16.4)	41,074 (32.5)	126,375
Daycare is useful for cognitive development of a child	25,923 (20.5)	39,463 (31.2)	36,795 (29.1)	24,247 (19.1)	126,428
Children only dance and sing at daycare, they do not learn anything	29,061 (23.0)	20,164 (16.0)	45,318 (35.9)	31,650 (25.1)	126,193
Helps to learn socialization	20,730 (16.4)	43,068 (34.1)	31,279 (24.8)	31,065 (24.6)	126,142
Daycare is not helpful for preventing injuries	884 (0.7)	2,791 (2.2)	74,956 (59.3)	47,708 (37.8)	126,339
We can also learn many things from our children who attend daycare	24,821 (19.8)	36,506 (29.1)	37,203 (29.7)	26,957 (21.5)	125,487
Overall environment of daycare is not good	1,046 (0.8)	2,094 (1.7)	76,374 (60.8)	46,108 (36.7)	125,622
Feel relaxed when child attends daycare	25,406 (20.3)	35,092 (28.0)	31,809 (25.4)	32,904 (26.3)	125,211
Creche mother beats/ yells at child	757 (0.6)	2,794 (2.2)	80,824 (64.4)	41,142 (32.8)	125,517
Have difficulties dropping off/ picking up child from daycare	27,469 (21.8)	50,232 (39.8)	32,692 (25.9)	15,919 (12.6)	126,312

Creche mother/assistant cannot manage children in daycare	629 (0.5)	2,346 (1.9)	83,495 (66.5)	39,088 (31.1)	125,558
Toys/books used are not suitable for children	815 (0.7)	1,949 (1.6)	80,292 (63.9)	42,535 (33.9)	125,591
Children fight with each other at daycare	887 (0.7)	9,590 (7.6)	80,330 (64.0)	34,789 (27.7)	125,596
Rich and poor children are treated differently	403 (0.3)	1,032 (0.8)	67,877 (53.9)	56,572 (44.9)	125,884
Children face difficulties using toilet	2,152 (1.7)	10,047 (8.0)	80,677 (64.2)	32,857 (26.1)	125,733
No one feeds child when they are hungry	396 (0.3)	1,679 (1.3)	84,091 (67.0)	39,310 (31.3)	125,476
Child feels disturbed to sleep at noon	1,420 (1.1)	5,694 (4.5)	82,539 (65.6)	36,233 (28.8)	125,886

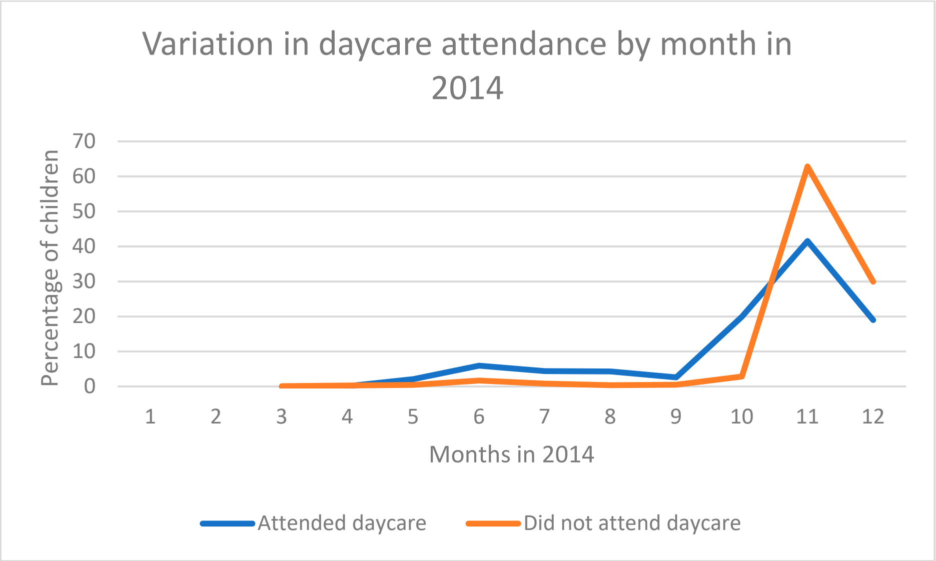

(a)

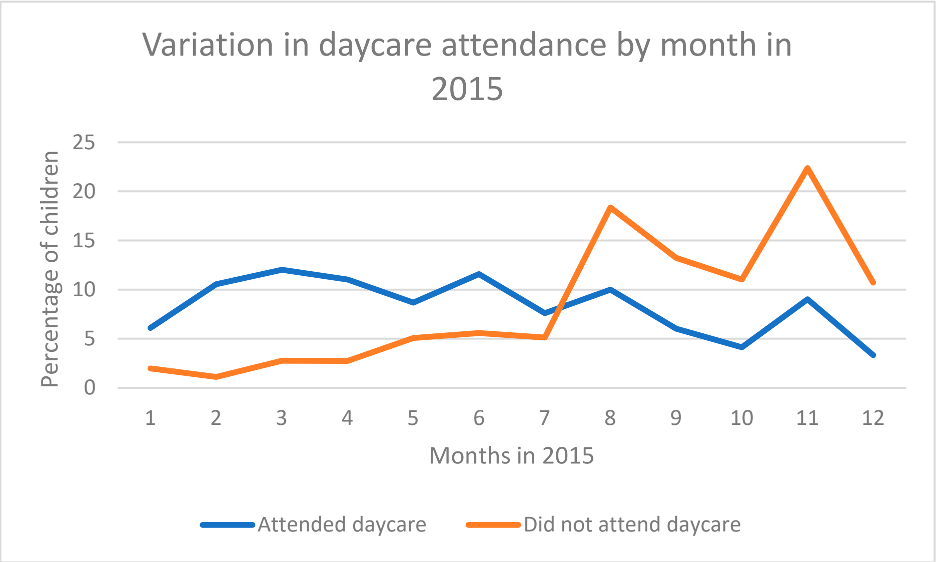

(**b**)

Supplementary Material, Figure S1. (a): Variation in daycare attendance by month in 2014, (b) Variation in daycare attendance by month in 2015.
